# A Novel Image Encryption Scheme Based on Collatz Conjecture

**DOI:** 10.3390/e20120901

**Published:** 2018-11-25

**Authors:** Dora M. Ballesteros, Jimmy Peña, Diego Renza

**Affiliations:** Telecommunications Engineering, Universidad Militar Nueva Granada, Carrera 11 No. 101-80, Bogotá 110111, Colombia

**Keywords:** image encryption, Collatz conjecture, ciphered audio, scrambling, diffusion

## Abstract

Image encryption methods aim to protect content privacy. Typically, they encompass scrambling and diffusion. Every pixel of the image is permuted (scrambling) and its value is transformed according to a key (diffusion). Although several methods have been proposed in the literature, some of them have been cryptanalyzed. In this paper, we present a novel method that deviates the traditional schemes. We use variable length codes based on Collatz conjecture for transforming the content of the image into non-intelligible audio; therefore, scrambling and diffusion processes are performed simultaneously in a non-linear way. With our method, different ciphered audio is obtained every time, and it depends exclusively on the selected key (the size of the key space equal to $8.57\times 10^{506}$). Several tests were performed in order to analyze randomness of the ciphered audio signals and the sensitivity of the key. Firstly, it was found that entropy and the level of disorder of ciphered audio signals are very close to the maximum value of randomness. Secondly, fractal behavior was detected into scatter plots of adjacent samples, altering completely the behavior of natural images. Finally, if the key was slightly modified, the image could not be recovered. With the above results, it was concluded that our method is very useful in image privacy protection applications.

## 1. Introduction

Nowadays, the amount of information posted on public sites or transmitted by digital form is huge. For example, the quantity of image uploads every day on Facebook is higher than three hundred million. Most times, the images are not content-sensitive, so it is not important if they are public; however, in other cases, the owner can wish to protect the privacy of its content. One way to provide privacy to images is through an encryption scheme [1], which has the objective of transforming the (secret) image to an unintelligible form to mask its perceptual content. The posted image can look like a noisy image, and only the authorized destination user can reveal the secret content.

In general, an encryption process encompasses two parts, the first one is related to a permutation task (confusion) and the second one to diffusion [2,3]. The aim of the first stage is to place out every pixel of the image in another position, whereas its value is changed in the diffusion process. The histogram of the original image (i.e., plain text) changes completely in the encrypted image [4]. It is expected that the histogram of the encrypted image looks like uniform distribution and its entropy will be the highest possible [5,6].

In the literature, the major effort in image encryption schemes has been providing security in terms of permutation/diffusion generation. In the last several years, chaotic sequences have been widely used [7,8,9,10,11,12]. G. Ye in 2010 [7] proposed an image scrambling encryption algorithm based on permutations of the pixel binary values in the image, by columns and rows, according to a chaotic sequence. Huang proposed in 2012 [8] a chaotic image encryption algorithm based on a Chebyshev function. In the same year, Wang et al. [9] presented a chaotic system to encrypt the RGB bands of color images, showing correlation values between adjacent pixels in the encrypted images around 0.01. Zhou et al. [10] proposed in 2014 a scheme of two existing one-dimensional (1D) chaotic maps. Its advantage relies on the generation of a completely different encrypted image every time because of the seed maps (key). G. Ye and X. Huang proposed in 2016 [11] a solution to obtain keys from ECG signals and an auto blocking method to provide automatic assignment. Later, in 2017, Pak and Huang [12] proposed a scheme that uses two sine maps in the permutation step, with better results than those obtained in [9]. However, those schemes have been cryptanalyzed [13,14,15,16]. For example, Tu et al. in 2013 [13] presented a theoretical analysis and experimental simulation for recovering the original image from the encrypted image, for the method presented in [9]. Wang, Luan, and Bao [14] in 2014 carried out the chosen-plain text attack to the method of [8]. C. Li, D. Lin and J. Lü in 2017 [17] proposed an efficient known-plaintext attack and a general chosen-plaintext attack on the algorithm ISEA of the method in [7]. In a similar way, Dhall et al. in 2018 [15] demonstrated that differential cryptanalysis with linear equations allows one to discover the original images for the method proposed by [10]. Wang et al. [16] in 2018 broke the method proposed by [12]. C. Li, D. Lin, J. Lü, and F. Hao [18] recently published a summary of security defects of the algorithm proposed in [11]. Finally, Erick Yong Xie et al. in 2017 [19] provided some bases for further optimizing the attack on one of the well-known image encryption methods titled Fridrich’s scheme.

A second group of image encryption methods includes deoxyribonucleic acid (DNA) encoding before the process of scrambling and ciphering [20,21]. In terms of the randomness of the encrypted image, the results are similar to that obtained by chaotic sequences, and again this kind of method has been cryptanalyzed (e.g., [22]). Alternative solutions use cellular automata to perform the confusion and diffusion tasks suitable for parallel computing [23]. In other works, the tasks are performed in the transform domain [24].

The above approaches follow a traditional design. The permutation task is carried out, and the diffusion process is then applied. Although some proposals have simultaneously combined them [25], the structure of the encryption process has not been changed. Therefore, they are sensitive to being broken.

In order to provide a novel solution to transform an image into an unintelligible content, we have proposed a scheme with the following characteristics:

- The permutation and diffusion processes are replaced by an encoding block which uses a non-fixed length mapping.
- The encoding process is accomplished by following the Collatz conjecture.
- The encrypted content corresponds to a speech signal instead of a ciphered image.
- Security of the scheme relies only on the key, with a size of the key space equal to $8.57\times 10^{506}$.
- The process is completely reversible and highly sensitive to the key.

The rest of the paper is organized as follows. Section 2 provides a background of concepts related to the proposed scheme as well as metrics of performance measurement. Section 3 presents the proposed solution divided into two modules: image coding and image recovering. Section 4 illustrates the performance of the method with some examples. Section 5 provides the results of several simulations in terms of the measurement parameters. Finally, the research is concluded in Section 6.

## 2. Background of Concepts

### 2.1. Collatz Conjecture

The Collatz conjecture is a mathematical problem also called the 3x+1 mapping with the following hypothesis: for any integer number, there is a specific number of iterations that can reduce the number to one, by

(1) $$\begin{array}{c} T\left(x\right)=\left\{\begin{array}{ccc} x/2 & \mathrm{if} & x\;\mathrm{is}\;\mathrm{even}\\ 3x+1 & \mathrm{if} & x\;\mathrm{is}\;\mathrm{odd} \end{array}\right. \end{array}$$

with T(x) being the next value, and the applied operation is related to the type of the input number (even or odd). Although that conjecture has not been demonstrated theoretically, several documents have proved their truthfulness for small numbers [26,27].

For example, suppose that the input number is 3. Then, the first operation is 3x+1 because this number is odd. The result is 10. Now, the value 10 is divided by two, because that is an even number. The result is 5. Applying the corresponding rule, the number 16 is obtained. A division by two is applied, and the result is 8. With another iteration, the value of 4 is found. Again, a division by two is applied and the result is 2.

With the final iteration, the value 1 is reached. For this example, 5 iterations are needed to reduce the number 3 to 1 with the rules presented in Equation (1).

A curious peculiarity of the Collatz conjecture is the variable number of iterations to reach the number 1. In addition, this number of iterations can increase or decrease with large or small numbers. Therefore, the number *x* can use *m* iterations to reach the number 1, while the number x+1 can use *n* iterations, with n<m. This behavior is appreciated for data encoding [28,29]. This is explained in detail in Section 3.1.

### 2.2. Correlation Coefficient

This parameter is very useful to compare the entire image or the inter speech signal behavior. Its aim is to measure the level of linear correlation (similarity) between a pixel with its neighbors (diagonal, horizontal, or vertical) or between a sample with its neighbors (left or right). A natural image or speech signal is expected to have a high value of correlation coefficient, i.e., close to one. Otherwise, an encrypted image (or audio) tends to have this value very close to zero.

In the case of adjacent pixels or samples, the correlation coefficient is calculated by

(2) $$r_{A,B}=\frac{cov\left(A,B\right)}{\sigma_{A},\sigma_{B}}$$

where $\sigma_{A}$ and $\sigma_{B}$ are the standard deviation of *A* and *B*, respectively. *B* is the image obtained with the right (or left, diagonal, or down) neighbors of *A*. In the case of audio, *B* is the right sample of *A*. For example, for an image of 512×512 pixels, *A* is 512×511 pixels, with the last column of the original image discards; *B* is 512×511 pixels, with the first column of the original image discard. For an audio, *A* encompasses the 1 to *N* samples, while *B* encompasses the 2 to *N* samples.

### 2.3. Entropy

In the field of theory of communication, entropy plays an important role to measure the information content and redundancy. For digital systems in which the content is expressed in bits, the suggested way to calculate it is by means of Shannon’s entropy:

(3) $$H\left(x\right)=-\sum P\left(x_{i}\right)\log_{2}\left(P\left(x_{i}\right)\right)$$

where *x* is the input, and $P(x_{i})$ is the probability of occurrence of the value $x_{i}$.

If all the symbols are equally likely, i.e., if $P\left(x_{1}\right)=P\left(x_{2}\right)=\ldots =P\left(x_{n}\right)=1/n$ for *n* possible outcomes, entropy is maximal and it is equal to *n*. On the other hand, if, of *n* possible results, only one symbol is the outcome, i.e., if $P(x_{1})=1$ and $P(x_{i})=0$ for i=[2n], entropy is the lowest and is equal to 0. Therefore, entropy is a measure of the uncertainty of data; high uncertainty (i.e., equally likely data) corresponds to high entropy, and vice versa. A detailed analysis is presented in [30].

In the current case, our data correspond to gray-scale images, i.e., 256 possible outcomes. If all pixels are equally likely, the entropy of the image is 8. For speech signals quantized with 16-bits, the maximum entropy is 16 for uniform histograms.

### 2.4. Disorder Scrambling (DS)

Typically for image encryption, one parameter used for measuring the correlation between adjacent pixels is the gray difference degree (GDD), which calculates the gray difference of current pixel of both the original image and the encrypted image. However, in the current proposal, the encrypted output is not an image but an audio file with a different quantity of bits with respect to the original image. Therefore, GDD is not a feasible measurement for our scheme. Instead of that, we selected the parameter of disorder scrambling (DS), which has been used in other works of speech scrambling [28,31,32], through the formula:

(4) $$DS=\frac{\sum_{i=2}^{m-1}\sqrt{\left|x_{i}-x_{i-1}\right|+\left|x_{i}-x_{i+1}\right|}}{m-2}$$

where $x_{i}$ is the current sample of the audio, $x_{i-1}$ is the left sample of $x_{i}$, $x_{i+1}$ is the right sample of $x_{i}$, and *m* is the total number of samples. For natural audio signals, the value of DS is close to zero; for ciphered audio signals, DS is close to two (i.e., for speech signals in the range [−1,1]).

### 2.5. Structural Similarity Index (SSIM)

This parameter evaluates the similarity of a test image *x* with respect to a reference image, named *y*. Similarity is computed by the analysis of the luminance term (*l*), the contrast (*c*), and the structural term (*s*) defined as follows:

(5) $$l\left(x,y\right)=\frac{2\mu_{x}\mu_{y}+C_{1}}{\mu_{x}^{2}+\mu_{y}^{2}+C_{1}}$$

(6) $$c\left(x,y\right)=\frac{2\sigma_{x}\sigma_{y}+C_{2}}{\sigma_{x}^{2}+\sigma_{y}^{2}+C_{2}}$$

(7) $$s\left(x,y\right)=\frac{\sigma_{xy}+C_{3}}{\sigma_{x}\sigma_{y}+C_{3}}$$

where $\mu_{x}$ and $\mu_{y}$ are the mean of the input images, $\sigma_{x}$ and $\sigma_{y}$ are their standard deviation, and $C_{1}$, $C_{2}$, and $C_{3}$ are constants.

Then, in a general form, SSIM is obtained, according to:

(8) $$SSIM\left(x,y\right)=l\left(x,y\right)*c\left(x,y\right)*s\left(x,y\right).$$

However, if $C_{3}=0.5*C_{2}$, then the above equation is rewritten as

(9) $$SSIM\left(x,y\right)=\frac{\left(2\mu_{x}\mu_{y}+C_{1}\right)\left(2\sigma_{xy}+C_{2}\right)}{\left(\mu_{x}^{2}+\mu_{y}^{2}+C_{1}\right)\left(\sigma_{x}^{2}+\sigma_{y}^{2}+C_{2}\right)}.$$

If the value of SSIM between two images is close to 1, they are perceptually equal (i.e., a user cannot easily identify the difference between the images); otherwise, if the value is close to 0, the perceptual similarity is null (i.e., it is very easy to identify the differences between the images).

## 3. The Proposed Scheme

Our proposed scheme for image encryption deviates from the traditional way of transforming an image into an output with non-legible content. It is worth noting that the mapping process between the input and the output is not one to one (like in the classical structure). Therefore, a pixel is transformed not only in position and value; the size of the bit word to represent it also changes. This is the main difference between our proposal and others found in the literature for image encryption.

**Figure 1:**
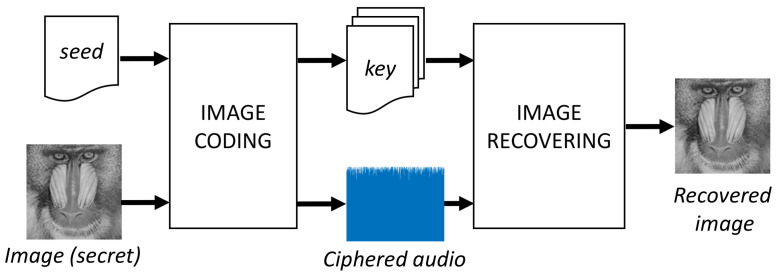
General block diagram of the proposed method.

Figure 1 shows the general architecture of the proposed solution, with two main modules: image coding and image recovering. Each module has blocks to perform the corresponding tasks.

### 3.1. Image Coding

The aim of this module is to transform the input image into audio with non-legible content. There is no relationship between the size of the image and the length of the audio, because it changes every time according to the input seed. The value of the seed is selected by the user.

**Figure 2:**
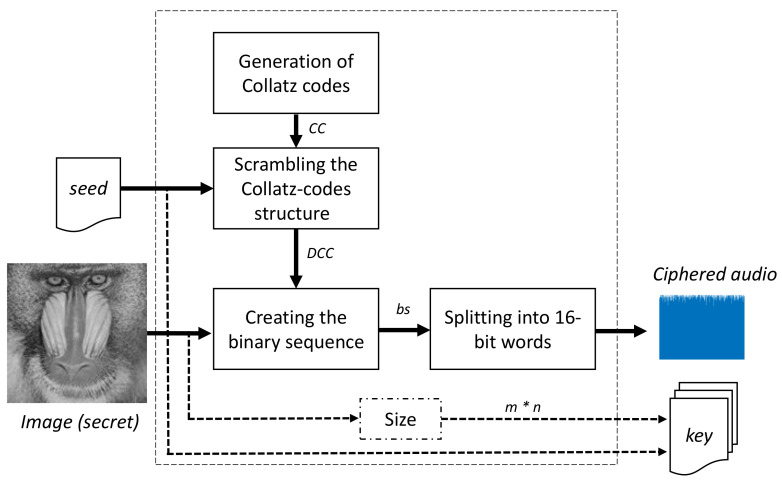
Specific block diagram of the image coding module.

**Figure 3:**
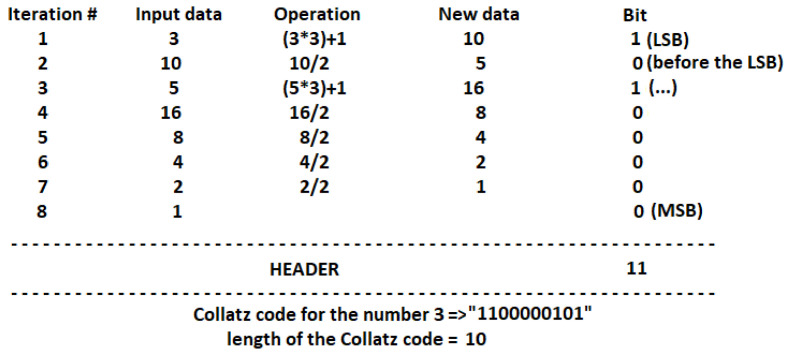
Example of Collatz code for the number 3.

**Figure 4:**
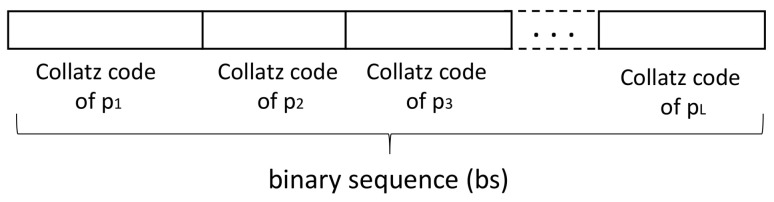
Example of the binary sequence.

Figure 2 presents the block diagram of this module. Each block is explained as follows:

- Generation of Collatz codes
  According to the Collatz conjecture explained in Section 2, we have proposed a new method for data encoding with variable output length as follows:
  - First iteration: if input data *x* is even, *x* is divided by two; a value of 0 is put in the LSB place. Otherwise, the operation 3x+1 is carried out; a value of 1 is put in the LSB place. If the result of the mathematical operation, $x_{a}$, is 1, the iteration process stops.
  - Second iteration: if $x_{a}$ is even, the operation $x_{a}/2$ is applied; a value of 0 is located in the position before LSB. Otherwise, the operation $3x_{a}+1$ is carried out; a value of 1 is put before the LSB place. If the result of the mathematical operation, xb, is 1, the iteration process stops.
  - The above procedure is performed until the value of 1 is reached. Its corresponding code is 0. Then, the iteration process stops.
  - In the last step, a header “11” is put at the beginning of the binary code. Therefore, the Collatz code length is equal to the number of iterations needed to reach the value of 1, plus the length of the header. Figure 3 illustrates an example.
  According to Figure 3, a 10-bit Collatz code is obtained for x=3. For the case x=4, only three iterations are required to reduce the value to 1, and its Collatz code length is then 5. On the other hand, for x=5, the number of iterations required is 6. Then, its Collatz code length is 8. It is clear that the Collatz code does not follow a “specific rule” in terms of its length, which means larger numbers can require a higher or lower number of iterations.
  Consequently, our proposed encoding method for image encryption has the following characteristics:
  1. The above coding method works for positive integer numbers.
  2. Since gray-scale images have their pixels in the range of 0 to 255, the value of the pixel is increased by 1 before applying the iterative process. This means our collection Collatz codes are in the range [1,256] instead of [0,255].
  3. The length of the Collatz code is not a fixed value. There is not a specific rule in terms of its length.
  4. Every code begins with the header “11” because this sequence is not viable with the proposed iteration process. Therefore, if a number is odd, the following number is always even, and the code corresponding to the sequence odd – odd (i.e., “11”) thus does not exist.
  At the output of this block, a cell array of 256 cells and variable number of elements in each cell is obtained. The first cell has the Collatz code of the number 1, the second cell has the Collatz code of the number 2, the last cell has the Collatz code of the number 256, and so on.
- Scrambling the Collatz-code structure
  The aim of this block is to provide a level of security of the encoding method, because if a non-authorized user knows the method, the image content can be revealed. Then, the cell array obtained in the above block is scrambled according to a seed. A new sequence is obtained, and every row of the structure is then located in a new position. Since the total number of sequences is $256!=8.57\times 10^{506}$, our system can work with a huge number of available scrambled matrices.
  The output of this block, DCC, is a cell array with similar characteristics to the one obtained in the last block. However, in this case, the first cell does not contain the Collatz code of the number 1. With a new seed, the corresponding code to a specific row changes every time.
- Creating the binary sequence
  Once the scrambled structure, DCC, is obtained, the next step consists in creating the binary sequence. This block is performed with the following steps:
  - The input image pixels sweep from left to right and top to bottom. The output of this step is a 1D sequence of L elements (with L=m×n, *m* is the number of rows, and *n* the number of columns of the image).
  - The first value, $p_{1}$, of the 1D sequence is selected. Its Collatz code corresponds to the row $p_{1}+1$ of the scrambled structure. For example, if $p_{1}$ is equal to zero, its Collatz code is the first row of DCC. This code is located at the beginning of the binary sequence, bs.
  - The second value, $p_{2}$, of the 1D sequence is selected. Its Collatz code corresponds to the row $p_{2}+1$ of the scrambled structure. Its code is located at the end of the binary sequence, bs.
  - The above procedure is repeated for the *L* elements of the 1D sequence (Figure 4).
- Splitting into words of *n* bits
  One important characteristic of our proposal is that the output of this module is an audio instead of a ciphered image. One of the reasons to change the format of the content is that the number of bits of the image differs from the number of bits of the encoded sequence. In addition, the relationship between secret and ciphered content is very low.
  Therefore, in this block, a task related to splitting the binary sequence into *w* blocks of 16 bits each is performed. In the case that the last block contains less than 16 bits, the rest of the sequence is set to zero. Later, every block of 16 bits is transformed to a floating point value in the range of −1 to 1. Finally, data are saved in a wav file.

### 3.2. Image Recovering

Two types of data are transmitted between the image coding module and the image recovering module: the ciphered audio (public information) and the private key. Two separate channels are used to transmit each one. For example, the ciphered audio is sent through WhatsApp and the private key is sent via e-mail. Once both data are obtained by the intended receiver, the process for recovering the original image is performed using the following blocks: the generation of Collatz codes, scrambling the Collatz codes, splitting them into words of variable length, and creating the recovered image.

**Figure 5:**
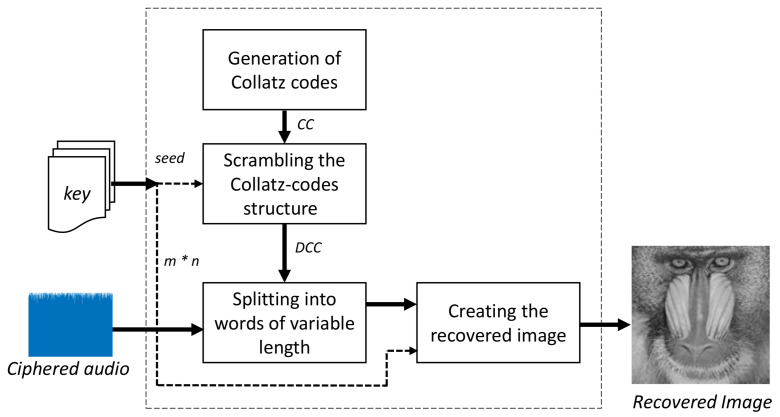
Specific block diagram of the image recovering module.

Figure 5 shows the block diagram of this module. Every block is explained as follows:

- Generation of Collatz codes
  This block works equally with the corresponding image coding module. Its aim is to obtain a structure of Collatz codes for the numbers 1 to 256.
- Disordering up the Collatz codes
  In a similar way to its counterpart of the image coding module, in this block, the above structure gets disordered in terms of its rows, according to the input seed.
- Splitting into words of variable length
  The input of this block is the ciphered audio. The first step consists in transforming the floating-point value of every sample into a binary code of 16 bits. Secondly, all codes are put together into a binary sequence of length *Z*, where *Z* is the result of multiplying the total number of samples by 16. Next, each header “11” is located in the above binary sequence. Finally, the binary sequence is split into frames (i.e., Collatz codes) taking into account the position of each header. The number of obtained codes is equal to the number of pixels of the secret image.
- Creating the recovered image
  In the last block of this module, every Collatz code is transformed to a decimal value in the range 0 to 255. The first code obtained with the above block is searched into the scrambled structure. Once a match is found, the position of the code minus one corresponds to the decimal value of the pixel. For example, suppose a code “110000101” is found in the first row of the structure. The value of this corresponding pixel is then zero. This procedure is carried out for every Collatz code. Once the decimal value of all pixels has been obtained, the last step consists in rearranging the pixels from left to right and top to bottom. The number of rows and columns of the image is included in the key, together with the value of the seed. The output of this block is the recovered gray-scale image.

## 4. Simulation Results

**Figure 6:**
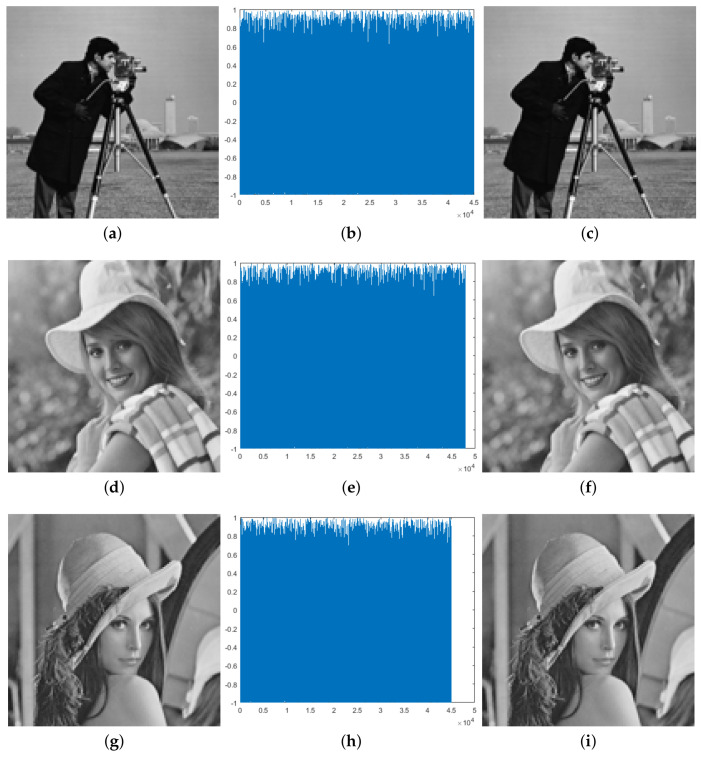
Preliminary results: Graphs (**a**,**d**,**g**) show original images; graphs (**b**,**e**,**h**) show cipher audios; graphs (**c**,**f**,**i**) show recovered images (available at https://data.mendeley.com/datasets/y8kn5mx4d2/draft?a=384e6a23-062e-401b-bdcd-621be1f952da).

This section provides some examples using the proposed scheme. Figure 6 shows the gray-scale test images (inputs), their cipher audios, and the corresponding recovered images.

According to Figure 6, perceptual similarity between original images and their recovered images is very high. This is confirmed with values of SSIM around 0.999. On the other hand, it is observed that all ciphered audio signals look like noise and are very similar between them, although they come from different images.

**Figure 7:**
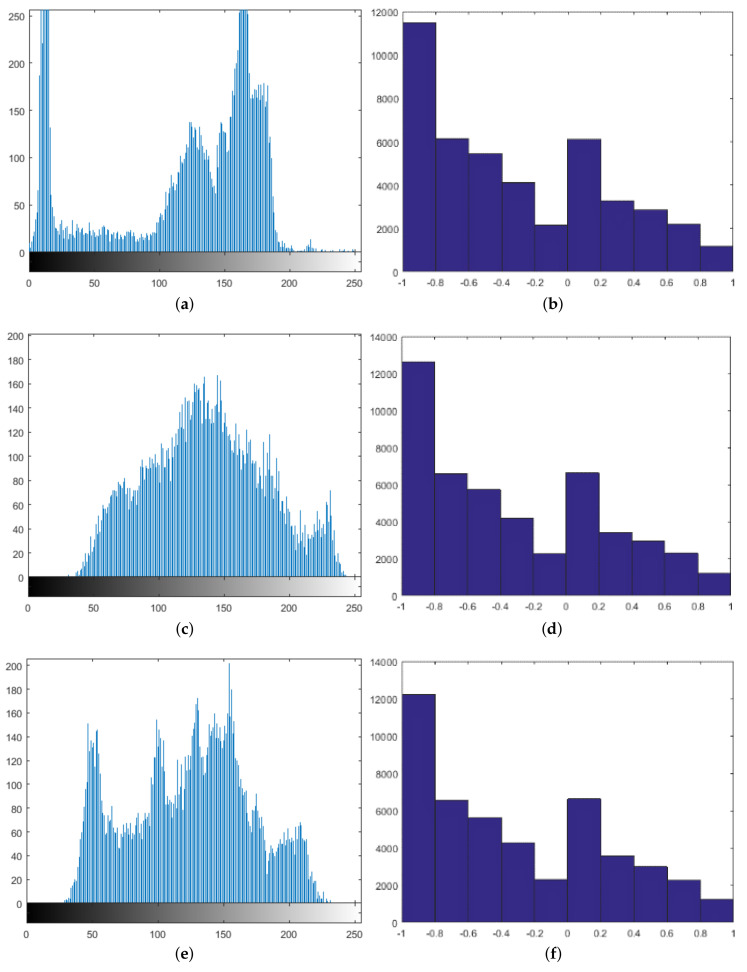
Preliminary results: Graphs (**a**,**c**,**e**) show histograms of original images; graphs (**b**,**d**,**f**) show a histogram of cipher audios.

Next, Figure 7 plots the histograms of the images and their ciphered audio signals. It can be seen that the behavior in terms of the histogram changes completely (available at https://data.mendeley.com/datasets/y8kn5mx4d2/draft?a=384e6a23-062e-401b-bdcd-621be1f952da). This topic will be discussed in more detail in Section 5.3.

## 5. Security Analysis

An important aspect to evaluate in any encryption scheme is related to its security analysis. Typically, this encompasses security key analysis, sensitivity to the changes of plain image, data correlation analysis, and information entropy analysis. For the following tests, we used 20 plain images and five keys per image, so 100 cipher audio signals were obtained.

### 5.1. Security Key Analysis

A good encryption scheme must provide a high level of security through its key. This aspect is evaluated in two parts: the size of the key space and key sensitivity analysis.

#### 5.1.1. Size of the Key Space

**Figure 8:**
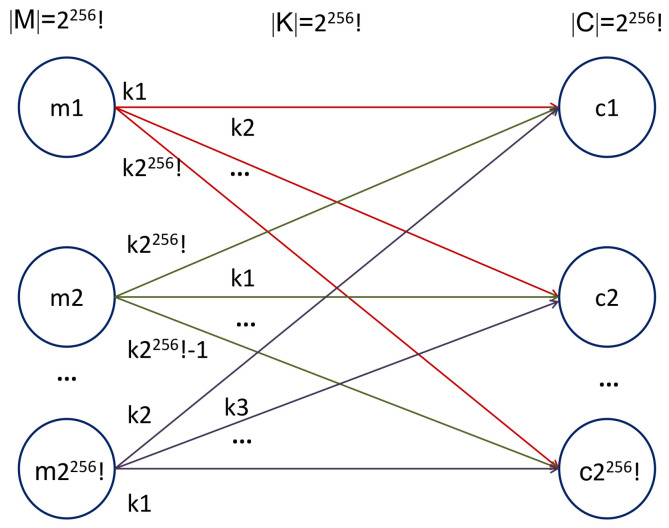
Analysis of perfect secrecy in our proposal.

In the paper titled “Communication Theory of Secrecy Systems,” Shannon defined the rules for unconditionally secure systems, working with *M* messages, *K* keys, and *C* cipher messages. One way to represent a secrecy system is a line diagram, in which the possible messages are represented in the left part (by circles), the cipher messages in the right part (by circles, too), and the keys used to obtain an encrypted message are represented by lines that join the original message with the cipher message (see Figure 5 of [33]). For the current case, message is a gray-scale image (8-bit, i.e., 256 possible values per pixel). Therefore, 256! circles are plotted in the left part of the line diagram, one circle by each value that the message can take. On the other hand, our system works with 256 different Collatz codes; thus, 256! circles are plotted in the right part of the line diagram, one circle by each cipher message that can be obtained. Finally, the different ways to map the original pixel value to its code value are represented by lines (Figure 8). Since we have included the block “scrambling the Collatz-code structure” in the image coding module, the total number of possible mappings between the left and the right part of the line diagram is equal to 256!, so the size of the key space is $8.57\times 10^{506}$.

According to the above, our system satisfies:

(10) $$\left|K\right|=\left|M\right|=\left|C\right|$$

with $\left|K\right|$, $\left|M\right|$, and $\left|C\right|$ representing the size of the key space, the message space, and the cipher space, respectively. This satisfies the condition of perfect secrecy defined by Shannon.

#### 5.1.2. Key Sensitivity Analysis

**Figure 9:**
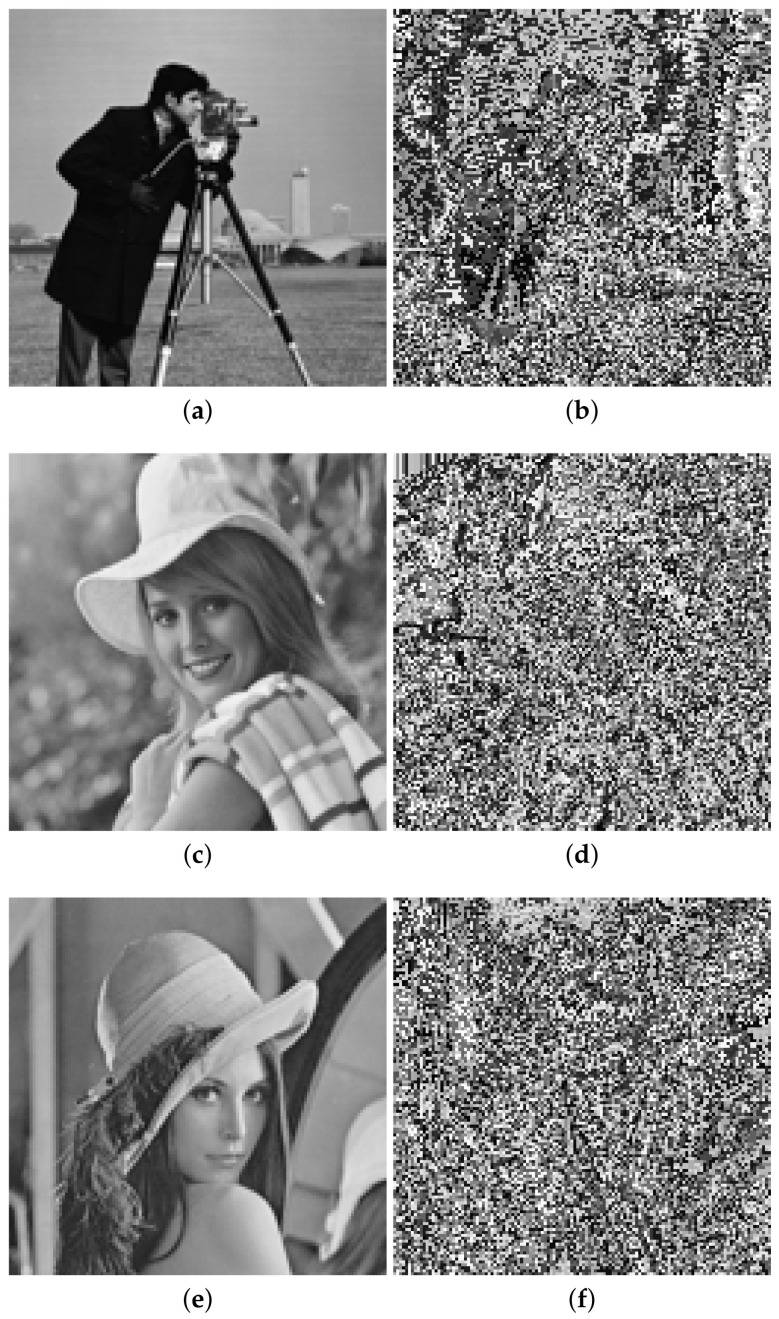
Key sensitivity analysis: Graphs (**a**,**c**,**e**) show original images; graphs (**b**,**d**,**f**) show their recovered images with a slightly different key.

A second analysis related to the key consists in making a slight change in the key within the image recovering module. Therefore, the key used to cipher the image is slightly different to the key used to decipher the image. Figure 9 shows an example of this test (available at https://data.mendeley.com/datasets/y8kn5mx4d2/draft?a=384e6a23-062e-401b-bdcd-621be1f952da). The image is ciphered with the key “Shannon” and deciphered with the key “shannon.” Although only the capital letter of the letter S was changed, the recovered image is perceptually different to the original one.

The above procedure was repeated for the 100 cipher audios, and in all cases the similarity between the original and the recovered image is perceptually null.

### 5.2. Sensitivity to the Changes of Plain Image

An ideal encryption scheme must be very sensitive to changes of the plain image. Therefore, if a pixel of the original image (plain image) is modified, the output will be significantly different. Sensitivity is directly related to the ability to resist differential attack. The more sensitivity, the greater the robustness against the attack.

One way to evaluate the ability to resist differential attack is with the UACI (Unified Average Changed Intensity) parameter, which compares two encrypted data of the same size obtained with the same key, but their original images differ in only one bit, as follows:

(11) $$UACI=\frac{\sum \left|C_{1}-C_{2}\right|}{L}*100$$

where $C_{1}$ and $C_{2}$ are Ciphered Audio 1 and 2, respectively; *L* is the total number of bits of the ciphered data. The UACI value is calculated on the binary sequence bs of Figure 2, and it was adapted of the original form applied to encrypted images.

After 100 tests, it was found that the value of UACI is between 0.44 to 0.46 with 95% of confidence.

### 5.3. Data Correlation Analysis

Our proposed system differs from traditional schemes in the fact that the output is not an encrypted image but an encrypted audio. This analysis is focused not on the image but on the audio. The purpose is to analyze if a sample of the audio is correlated to its neighboring samples (left or right) and to obtain a mathematical value of this correlation.

**Figure 10:**
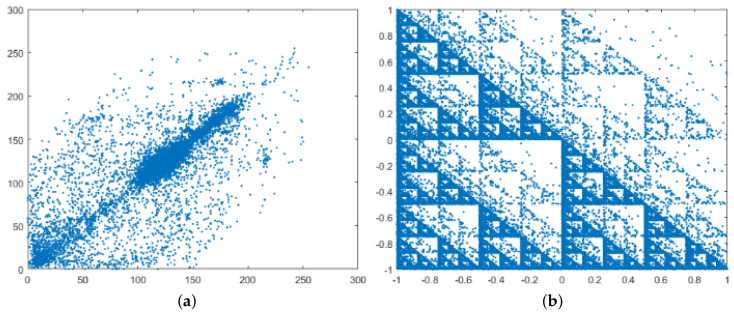

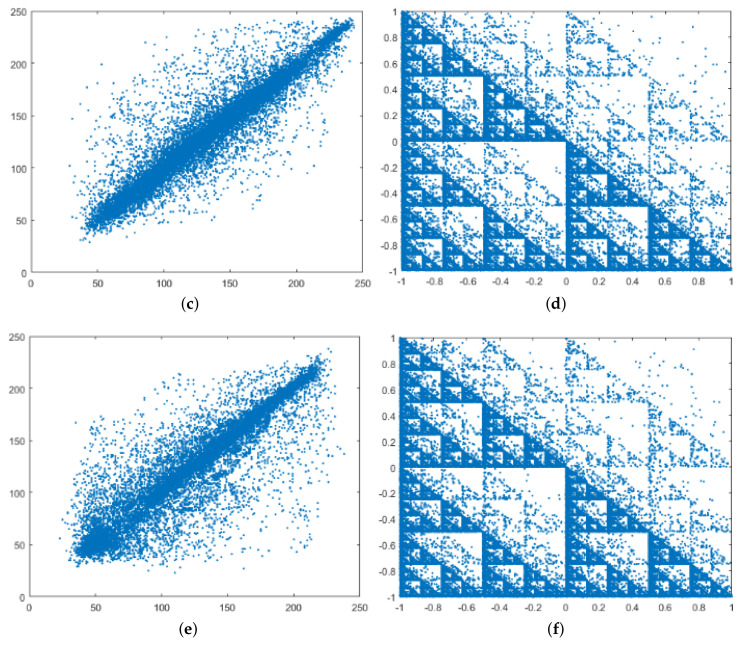
Correlation of adjacent pixels. Graphs (**a**,**c**,**e**) show the distribution of horizontal adjacent pixels of the images shown in Figure 7a,c,e. Graphs (**b**,**d**,**f**) show the distribution of adjacent samples of the cipher audio signals shown in Figure 7b,d,f.

Figure 10 shows plots of adjacent pixels of the plain images and the adjacent samples of their ciphered audios. It should be noted that in natural images or audio signals, this graph is a set of points around the main diagonal, but with our ciphered audios, a kind of fractal is found. This special behavior is always found even for different images or keys (plots are available at https://data.mendeley.com/datasets/y8kn5mx4d2/draft?a=384e6a23-062e-401b-bdcd-621be1f952da).

To specifically calculate the correlation between adjacent samples, Equation (2) is applied. For natural audios, this value is close to 1. Fo ciphered audio with non-intelligible content this value is close to 0.

### 5.4. Uncertainty and Disorder Analysis

**Figure 11:**
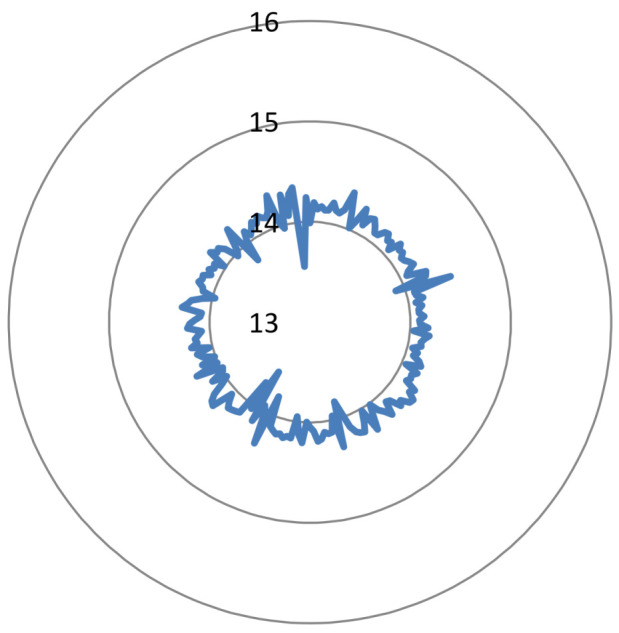
Radar plot of entropy: 100 values.

**Figure 12:**
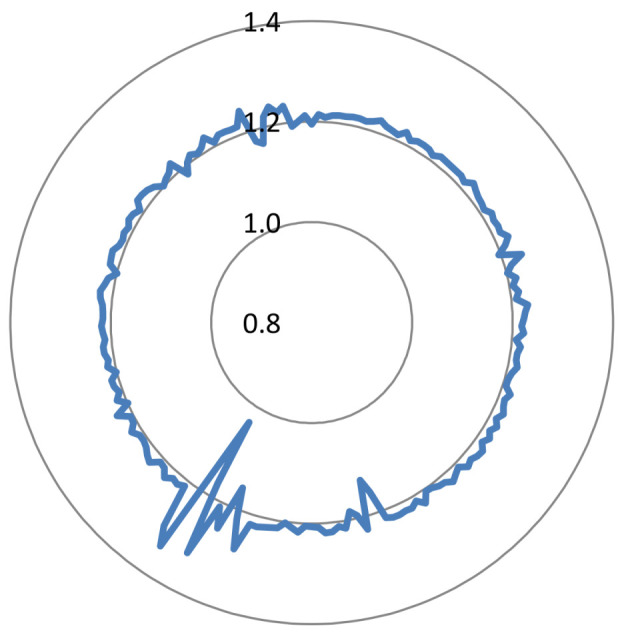
Radar plot of DS: 100 values.

This evaluation is focused on the entropy and DS of the ciphered audios. From the 100 audio signals under study, we obtained the results of Figure 11 and Figure 12.

According to Figure 11, all audio signals have entropy higher than 13 and around 14. Therefore, the uncertainty level of the ciphered signals is close to the maximum (i.e., 16 for audio signals with 16 bits/sample). In terms of DS (Figure 12), the ciphered signals obtained with our proposed method are very close to the maximum value (i.e., 2). All data are higher than 0.8, and most of them are close to 1.2. It is important to remark that the value of DS is much higher than other obtained in the literature for scrambling audio signals. For example, in [28], the value of DS was around 0.6.

Taking into account the above results of entropy and DS, it was concluded that our ciphered signals deviates from the characteristics/behavior of the images that they come from, and a behavior very close to the maximum uncertainty and randomization is obtained.

## 6. Comparison with State-of-the Art Methods

In this section, the proposed method and some image encryption methods (published in the last five years) are compared (Table 1). The metrics selected to apply the comparison are the size of the key space, key sensitivity, relative entropy, and UACI. The size of the key space is related to the security of the system. The higher the size of the key space is, the greater the effort to break the security of the system is. Key sensitivity is related to the response of the system to a very low change in the key, and it is measured in a perceptual way, through the deciphered image. Relative entropy is obtained as the ratio between the entropy of the encrypted data and the maximal entropy. Finally, UACI is related to the differential attack, and it represents the quantity of bits changed in the encrypted image when one bit of the original image changes; the higher the value of UACI is, the more robust the system is.

According to the results, the main advantage of our proposal over other methods lies in its security in terms of the size of the key space to resist brute force attack and inthe value of *UACI* to resist differential attack. All the analyzed methods have excellent results in terms of key sensitivity. This parameter has thus been completely satisfied. In terms of entropy, our proposal has lower relative entropy than other methods; however, there is a special behavior in our method found in the scatter plots (adjacent samples) of the encrypted data. A kind of fractal in these plots is obtained, with a completely different behavior than schemes based on chaotic maps, for example. This result is a particular characteristic of our proposal, which is unique in the state of the art.

## 7. Conclusion

Most of the state-of-the-art image encryption methods have reached the quasi-maximal value of entropy in the encrypted data, which is an excellent characteristic of this kind of systems. However, as far as we know, none of those methods have satisfied the principle of perfect secrecy of Shannon, which claims that a system is unconditionally secure if the size of the key space is equal to the size of the message space; for this reason, it is expected that in the near future those methods can be broken. In this paper, we have proposed an image encryption scheme that differs from recent state-of-the-art methods in the way to obtain the encrypted data; we have combined the diffusion and confusion tasks by means of a variable length coding of the pixels based on the Collatz conjecture, and not by chaotic sequences. Our proposal satisfies the principle of secure secrecy of Shannon’s theory, which means our system is the most robust scheme against brute force and differential attacks of the state-of-the art methods. One aspect to improve in our method lies in the entropy value of the encrypted data. However, the behavior of the original data is completely modified in its encrypted version, i.e., what can be verified, for instance, by looking at the histograms and the plots of adjacent data. Regarding this last point, fractal behavior has been found in the encrypted data, which is a distinctive pattern of our method.

According to the above analysis, we propose the following themes for future work:

- Identify weaknesses of our proposal in terms of the probability of the available space (theoretical it is equally likely) and what can affect the robustness against brute force attack and differential attack. This is related to the way to randomize the Collatz codes. For that purpose, three pairs of original images and encrypted data are available at https://data.mendeley.com/datasets/y8kn5mx4d2/draft?a=384e6a23-062e-401b-bdcd-621be1f952da.
- Apply bit scrambling to the binary sequence, bs, of Figure 2 with the purpose of increasing the entropy of the encrypted data.
- Explore other choices of image coding based on the Collatz conjecture. For example, applying the Collatz code not for the pixel value but for the pixel position. Analyze the performance of the system in terms of security.

## Figures and Tables

**Table 1:** Performance of some methods of image encryption.

Ref.	Method	Size of the Key Space	Key Sensitivity	Relative Entropy	*UACI*
[34]	Chaotic maps	$10^{56}$	Very high	99.8%	0.5 (binary images) 0.33 (gray-scale image)
[35]	Chaotic maps	$2^{256}=1.1\times 10^{77}$	Very high	99.7%	0.33
[36]	DNA encoding + chaos	$10^{93}$	Very high	99.8%	0.33
[37]	Chaotic maps	$10^{210}$	Very high	99.8%	0.33
[38]	DNA encoding + chaos	$3.4\times 10^{38}$	Very high	99.9%	0.33
ours	Collatz encoding	$8.57\times 10^{506}$	Very high	87.5%	0.44
